# Nonlinear ionization dynamics of hot dense plasma observed in a laser-plasma amplifier

**DOI:** 10.1038/s41377-020-00424-2

**Published:** 2020-11-18

**Authors:** F. Tuitje, P. Martínez Gil, T. Helk, J. Gautier, F. Tissandier, J.-P. Goddet, A. Guggenmos, U. Kleineberg, S. Sebban, E. Oliva, C. Spielmann, M. Zürch

**Affiliations:** 1 Institute for Optics and Quantum Electronics, Abbe Center of Photonics, University of Jena, Jena, Germany; 2 Helmholtz Institute Jena, Jena, Germany; 3 Departamento de Ingeniería Energética and Instituto de Fusión Nuclear “Guillermo Velarde”, ETSI Industriales, Universidad Politécnica de Madrid, Madrid, Spain; 4 Laboratoire d’Optique Appliquée, ENSTA Paris, Ecole Polytechnique, CNRS, Institut Polytechnique de Paris, Palaiseau, France; 5 Department for Physics, Ludwig-Maximilian-University Munich, Garching, Germany; 6 UltraFast Innovations GmbH, Garching, Germany; 7 Fritz Haber Institute of the Max Planck Society, Berlin, Germany; 8 Department of Chemistry, University of California at Berkeley, Berkeley, CA USA; 9 Materials Sciences Division, Lawrence Berkeley National Laboratory, Berkeley, CA USA

**Keywords:** X-rays, Laser-produced plasmas, High-harmonic generation, Nonlinear optics

## Abstract

Understanding the behaviour of matter under conditions of extreme temperature, pressure, density and electromagnetic fields has profound effects on our understanding of cosmologic objects and the formation of the universe. Lacking direct access to such objects, our interpretation of observed data mainly relies on theoretical models. However, such models, which need to encompass nuclear physics, atomic physics and plasma physics over a huge dynamic range in the dimensions of energy and time, can only provide reliable information if we can benchmark them to experiments under well-defined laboratory conditions. Due to the plethora of effects occurring in this kind of highly excited matter, characterizing isolated dynamics or obtaining direct insight remains challenging. High-density plasmas are turbulent and opaque for radiation below the plasma frequency and allow only near-surface insight into ionization processes with visible wavelengths. Here, the output of a high-harmonic seeded laser-plasma amplifier using eight-fold ionized krypton as the gain medium operating at a 32.8 nm wavelength is ptychographically imaged. A complex-valued wavefront is observed in the extreme ultraviolet (XUV) beam with high resolution. Ab initio spatio-temporal Maxwell–Bloch simulations show excellent agreement with the experimental observations, revealing overionization of krypton in the plasma channel due to nonlinear laser-plasma interactions, successfully validating this four-dimensional multiscale model. This constitutes the first experimental observation of the laser ion abundance reshaping a laser-plasma amplifier. The presented approach shows the possibility of directly modelling light-plasma interactions in extreme conditions, such as those present during the early times of the universe, with direct experimental verification.

From fusion dynamics in stars to terrestrial lightning events to new prospects for energy production^1,2^ or novel light sources^3–7^, hot dense plasmas are of importance for an array of physical phenomena^8,9^. Due to the plethora of correlations in highly excited matter, direct probing of isolated dynamics remains challenging. Here, the 32.8-nm emission of a high-harmonic seeded plasma amplifier^5^ is ptychographically imaged in the longitudinal direction in the extreme ultraviolet (XUV) region. In excellent agreement with ab initio spatio-temporal Maxwell–Bloch simulations, spatially limited overionization of krypton is observed. This constitutes the first experimental observation of the laser ion abundance reshaping a laser-plasma amplifier. The findings have direct implications for upscaling plasma-based XUV and X-ray sources and allow modelling light-plasma interactions in extreme conditions, such as those at the early times of the universe, with direct experimental verification.

Determining important plasma parameters, such as the electron density and temperature profile in a hot plasma, is of widespread importance, for instance, in nuclear fusion research, uniform shockwave formation in z-pinch experiments^2^ or stable confinement conditions in temperature-based fusion setups^1^. Table-top particle accelerators based on the plasma wake-field effect can be used to implement high-energy research at the lab scale and pave the way to easy-access particle and radiation sources for materials science or medicine, where optimized spatio-temporal ionization profiles in the host plasma lead to large acceleration gradients^10^. Furthermore, plasma-based radiation sources provide a wide array of radiation from the visible to the X-ray regime and enable applications from spectroscopy^11^ to lithography^12^. The properties of the radiation depend on the generation process of the plasma. Spatially and temporally highly coherent and directed radiation^5–7^, as well as diffuse incoherent illumination^13–15^, can be achieved. Understanding and classifying the inherent effects occurring in this extreme kind of matter is, therefore, a crucial part of exploring the surrounding nature or enhancing technology. While plasma generation is in most cases straightforward, analysing its composition is nontrivial. Due to the multitude of internal processes occurring (excitation, recombination, collision, etc.) and the turbulent nature of the gaseous media, the observation methods have to be highly adapted to the plasma conditions.

Here, the inner dynamics of a laser-pumped plasma channel and the induced spatial ionization structure are resolved with high fidelity. The experiments are performed with a table-top near-infrared (NIR) laser-driven soft-X-ray laser (SXRL) acting as a laser-plasma amplifier for a high-harmonic generation (HHG) seed pulse to obtain high spatial and temporal coherence and a synchrotron-like total flux^5^ (see Methods). In this system, eight-fold ionized krypton ions (Kr^8+^), pumped by collisions with free electrons of the plasma, act as an amplification medium resembling a nickel-like quasi-3-level laser scheme. This enables strong amplification in the XUV domain. The amplifying medium, consisting of highly ionized krypton atoms, is generated by an intense laser pulse. Hence, the ionization state is controllable by the laser and gas parameters. The seed, which experiences amplification, simultaneously acts as a probe of the laser-plasma interaction. Subsequently, ptychographic coherent diffraction imaging is employed to measure the coherent complex-valued emitted radiation field with high spatial resolution. The ptychographic reconstruction of an arbitrary object directly recovers the complex-valued wavefront^16^, which allows backpropagation to the laser-plasma amplifier. Four-dimensional Maxwell–Bloch simulations are used to model the amplification of the HHG beam throughout the plasma, i.e. in the forward direction, taking plasma dynamics and inhomogeneities into account. The simulated spatio-temporal laser-plasma amplifier outputs are in excellent agreement with the experimental observations. This enables a comprehensive view of the ionization dynamics in the laser-generated plasma and reveals insights into the ionization mechanisms. The results indicate that nonlinear ionization occurs in the amplifier.

The laser-plasma amplifier gain medium is a plasma of Kr^8+^ ions created via optical field ionization by an intense NIR femtosecond pulse^17^. Amplification of the HHG seed pulse occurs for the 3d^9^4d-3d^9^4p transition of the Kr^8+^ ion at 32.8 nm. Pumping of the population inversion between these two states is ensured by collisions with the hot electrons of the plasma, mainly from the ground state of the Kr^8+^ ions (Fig. 1a). The HHG seed spectrum can be tuned such that one harmonic of the seed pulse resonates with the lasing transition, enabling stimulated emission. During this coherent amplification process, specific characteristics of the laser-plasma amplification process depending on the lasing ion and electron density are imprinted on the seed pulse. See Methods for more details.

**Fig. 1:**
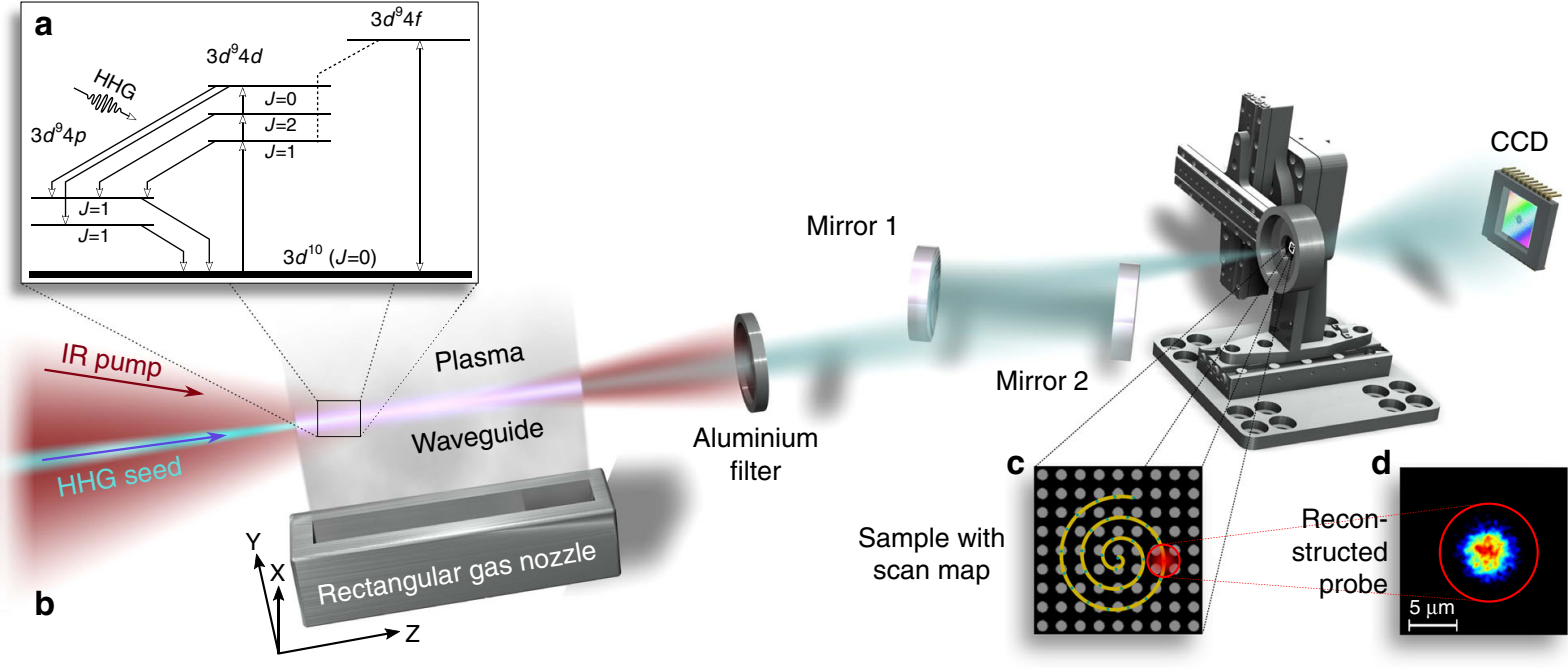
Experimental setup and operation scheme of the laser-plasma amplifier with diagnostics. **a** A series of infrared pump beams (see Methods) creates a plasma waveguide, excites nickel-like Kr^8+^ and creates population inversion of the 3d^9^4d state, forming a laser-plasma amplifier. Here, only dipole-allowed transitions involved in the amplification process are shown (level scheme inset^33^). The HHG seed at a 32.8 nm wavelength is coupled into the plasma channel and is amplified by stimulated emission (4d–4p transition in Kr^8+^). **b** Schematic setup of the experiment. **c** The emitted radiation is refocused using multilayer mirrors onto the sample consisting of a regular hole pattern (C depicts an SEM image). Ptychography is performed using a spiral scan pattern (indicated in yellow) with a CCD recording a coherent diffraction pattern at each scan point. The relation between the scan map and probe size is marked with a red circle. **d** The recorded diffraction patterns using ptychography can be reconstructed to retrieve the coherent complex-valued illumination function (probe) (**d** depicts the amplitude of the reconstructed illumination function)

The seeded laser-plasma amplifier output is imaged and demagnified onto a sample composed of a regular hole pattern by a system of two spherical mirrors (Fig. 1b; see Methods). The coherently diffracted light is then recorded in transmission geometry. To retrieve the complex-valued illumination field, further called the *probe*, ptychography is employed^16,18^. Compared to other wavefront sensing techniques, such as the use of a Hartmann–Shack sensor, ptychography enables higher spatial sampling, which is important for precise free-space backpropagation. Additionally, using an object with aperture sizes comparable to the focus size, a higher flux on the detector can be achieved, reducing the exposure time. Recovering the phase is crucial to enabling backpropagation to the source and, therefore, to analysing the plasma. To achieve a low reconstruction error, the object was scanned on a spiral path with 30 overlapping scan points (Fig. 1b). For further details, see Methods. At each scan position, five diffraction patterns were recorded, allowing subsequent averaging. The retrieved probe represents the coherent part of the full illumination field (amplitude depicted in Fig. 1c) and shows a diameter of 5.6 ± 0.2 µm based on the full width at half maximum (FWHM). With an emitting diameter of the plasma channel of 90 ± 10 µm and a demagnification of 10, 40 ± 10% of the beam area is spatially coherent, which is substantially higher compared to a nonseeded SXRL^6^ and similar to a free-electron laser^19^. Following the successful retrieval of the complex-valued illumination field in the sample plane, backpropagation to the exit plane of the plasma channel using the angular spectrum propagation method is performed. See Methods and supplementary materials Section S1 for more details.

The experimentally obtained complex-valued exit field of the laser-plasma amplifier is depicted in the inset of Fig. 2a central, local dip is observed in the radial intensity profile (Fig. 2, blue solid line). The radial phase profile (Fig. 2, red dashed line) shows a parabolic shape resulting from the hydrodynamic expansion of the plasma after ignition. Due to the sudden expansion^20^, the electron density radially increases, changing the refraction index accordingly. The increased standard deviation of the phase for larger radii emerges from the near-zero intensity due to the reconstruction process and the consequently random phases.

**Fig. 2:**
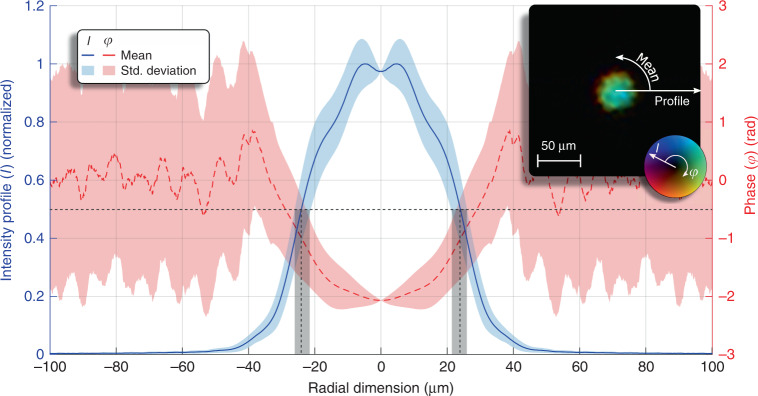
Reconstructed exit field of the laser-plasma amplifier. The complex-valued retrieved exit field of the laser-plasma amplifier is pictured in the inset. Here, the hue and brightness represent the phase and intensity, respectively. The radial profile of the intensity shows a Gaussian-like profile with a dip in the centre. Simulations indicate that an overionized zone in the laser-plasma amplifier leads to decreased amplification in the centre of the channel. The phase profile shows a parabolic shape caused by the radially decreasing refraction index. Note: the high standard deviation of the phase above a 40 µm radius arises from the low intensity and the corresponding random phases during the reconstruction. The diameter of the exit field of 52 ± 5 µm (FWHM) is marked with black dashed vertical lines, where the grey bar represents the error

To understand the complex plasma dynamics that result in the observed laser-plasma amplifier output, it is necessary to fully model the propagation of the HHG seed pulse within the plasma in three spatial dimensions. Additionally, due to the ultrafast nature of the seed pulses, the amplification process is nonadiabatic and requires appropriate time-domain modelling.

The amplification in the plasma is modelled with the 3D Maxwell–Bloch code *Dagon*^21^. This code solves the Maxwell wave equation for the electric field in paraxial form using the slowly varying envelope approximation (SVEA). This equation is enhanced with a constitutive relationship for the polarization and rate equations for the upper and lower level populations of the lasing transitions. These equations are derived from Bloch equations. The temporal evolution of the plasma after pumping is obtained from a collisional-radiative code, *OFIKinRad*^22^, and previous particle-in-cell (PIC) modelling^20,23^ with the *WAKE-EP*^24^ and *Calder-Circ*^25^ codes. The plasma waveguide profile was obtained from experimental results^5^ and hydrodynamic simulations^20^ with the code *ARWEN*^26^. For further information, see Methods and supplementary information S5.

Figure 3 shows the electron density profile of the plasma waveguide and the Kr^8+^ distribution along the laser-plasma amplifier. The NIR and HHG beams propagate from the upper to the lower part of the depicted waveguide. The steep rise in electron density at the bottom part of the figure marks the position of the NIR pump pulse, which creates the lasing ions by optical field ionization of the Kr^3+^ ions composing the waveguide. The lasing ion (Kr^8+^) profile after the NIR pump pulse traverses the amplifier is shown in Fig. 3b. A radial Gaussian profile is assumed for its abundance, with a flat-top region near the central part of the plasma, as given by PIC modelling. Taking the radial profile of the plasma waveguide into account results in a small parabolic structure near the central part of the channel. The central part of the amplifier appears to be overionized, according to PIC modelling. Focusing effects increase the intensity of the pump NIR beam in some regions of the amplifier, attaining the threshold to produce higher charged ions. Thus, Kr^8+^ is depleted in these regions, and the electron density is further increased. This result well fits the centre dip in the experimentally observed exit wave.

**Fig. 3:**
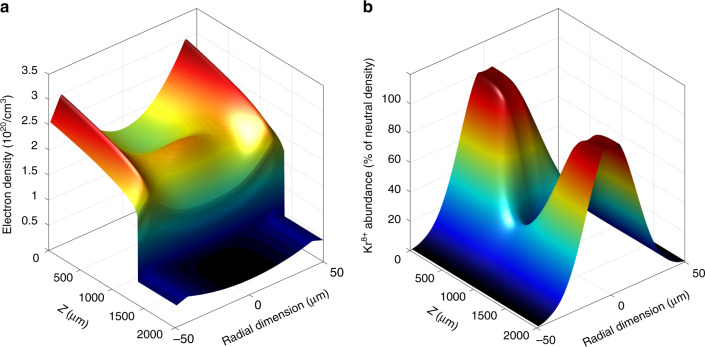
Spatial distributions of electrons and lasing ions in the amplifier following the NIR pump pulse. **a** Electron density profile in the plasma waveguide after propagation of the pump pulse to z = 1200 µm. **b** Lasing ion (Kr^8+^) abundance in the laser-plasma amplifier as a percentage of the neutral density after complete propagation through the channel. The lasing ion is depleted at Z = 1000 µm at the radial centre due to overionization. Thus, the electron density profile shows a corresponding peak in this region. Furthermore, **b** shows a groove of decreased ion abundance for r = 0 µm, resulting in an attenuated amplification, explaining the dip in intensity observed in the experiment (Fig. 2)

The spatio-temporal intensity distribution of the amplified HHG, as given by Maxwell–Bloch modelling, is shown in Fig. 4a. The duration of the pulse is a few hundred femtoseconds (<300 fs), in good agreement with previous experimental and modelling results^5^. The amplified HHG beam presents a rich structure, induced by the amplifier radial profile and its inhomogeneities. Temporal oscillations (Rabi oscillations between the 3d^9^4d and 3d^9^4p states) with a period of approximately 80 fs are clearly visible. In addition, the intensity iso-contours have a curved profile, induced by the radial distribution of the plasma waveguide. Instead of a single intensity maximum at the centre of the amplifier, two intensity maxima appear several micrometres from the central part. The parabolic shape of the plasma channel electron density along with the overionization in the channel reduces the amplification in the central part, resulting in the two-peak profile and the phase that the experiment revealed is in excellent agreement with the simulation, as shown in Fig. 4b.

**Fig. 4:**
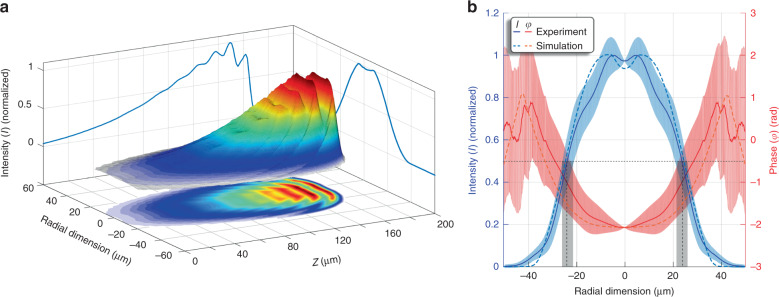
Spatio-temporal intensity profile of the amplified HHG pulse and comparison with experiment. **a** The beam shows a rich structure with temporal (Rabi) oscillations. The curved iso-intensity contours reveal two intensity maxima that are not located at the central part of the amplifier. All these structures are induced by the plasma waveguide inhomogeneous profile and the lasing ion abundance through its radial profile and the depletion of lasing ions in the central part of the amplifier. **b** The numerically accumulated intensity and phase show excellent agreement with the experimental results. Errors of experimental data are shown in pale colours, and the black bars indicate the diameter

In summary, ptychographic imaging is successfully employed to retrieve complex-valued illumination functions with high resolution and fidelity, and it is applied for the first time to imaging the plasma dynamics in a laser-plasma amplifier. While spatial filtering by the amplifier gain is usually expected to clean up the beam profile, a modulated wavefront is observed here, indicating an inhomogeneous distribution of the gain medium. Spatio-temporal calculations reveal the laser-plasma dynamics in the otherwise inaccessible high-density plasma channel. The simulation yields excellent agreement with the experimental observations, validating the parameters and models chosen to reproduce the complex ionization dynamics inside the waveguide. The observed inhomogeneous amplification is a consequence of the propagation of the required strong optical pump pulse. The accompanying nonlinear ionization results in a heterogeneous electron density, which directly correlates with the ionization degree of krypton. Overionization in the centre of the channel causes the Kr^8+^ abundance to decrease locally to 20% of the neutral density and results in an inhomogeneous amplification, which is imprinted on the output wave of the laser-plasma amplifier. More generally, the results indicate the limitations of upscaling laser-driven plasma amplifiers and related SXRL technologies based on optical field ionization and state boundaries for laser-based generation of hot dense plasmas with certain ion compositions. Furthermore, the observations reported here show the importance of four-dimensional modelling of the laser-plasma interaction, especially in highly ionized plasmas that can lead to substantial reshaping of all involved pulses. To our knowledge, this is the first observation where ab initio modelling correctly predicts the amplification in a laser-plasma amplifier. The experimental validation of the used models holds great promise for employing these numerical methods to predict future laser-plasma amplification schemes, as this method allows disentanglement of plasma shaping properties that are otherwise hidden. For example, the electron density inhomogeneities combined with the ion abundance of a certain ion state are hardly separable using, e.g. shadowgraphy or require a complex experimental setup supported by modelling, such as Thomson scattering techniques.

The observed Rabi oscillations in the laser-plasma amplifier lead to a strong modification of the temporal structure of the pulses that can be directly controlled by the optically prepared plasma. This approach of spatio-temporally modulating XUV pulses (see SI Section 6 for further simulations on spatial beam shaping) could open possibilities for, e.g. quantum computing^27^, where the ultrashort time-domain shaped pulses can be used for preparing the ion states of ultracold ions, fixed in optical lattices, and for switching between resonant core transitions, as the pulses are highly stable due to the fixed transition dipole moment. Furthermore, it opens up the possibility of tracking electronic^28^ and molecular^29^ dynamics in pump-probe experiments, where the controllable time-domain properties of the XUV pulse result in highly adaptable probe beams. Additionally, one could envision using the tuneable time-domain XUV pulse sequence as an exquisite source for XUV coherent spectroscopies. Using XUV radiation in this scheme opens a wide field for a plethora of studies regarding material selectivity.

## Materials and methods

### Experimental setup

The experiment was performed at Laboratoire d’Optique Appliquée (LOA) using the *Salle jaune* Ti:sapphire laser system^5^ able to deliver three independently compressed multiterawatt femtosecond pulses. The target was a high-density krypton gas jet equipped with a 5 mm long rectangular nozzle. It was pumped by a 1.4 J, 30 fs pulse focused by a 0.8 m focal length spherical mirror at an intensity up to 5 × 10^18^ W/cm^2^. Since the plasma electron density typically ranges from a few 10^19^–10^20^ cm^−3^, the pump pulse cannot propagate over more than a few hundred microns, so an optically preformed plasma waveguide^30^ was implemented. A 100 mJ, 30 fs “ignitor” pulse followed by a 700 mJ, 600 ps “heater” pulse was focused into the krypton jet using an axicon lens, resulting in a plasma column over the whole length of the gas jet. After hydrodynamic expansion of the plasma column^20^, a 5 mm long channel suitable for guiding the main pump pulse was achieved. After passing of the pump pulse, the plasma was mostly composed of Kr^8+^ ions and the hot electrons (E > 145 eV) needed to pump the population inversion^17^. The amplifier was seeded by an HHG pulse created by focusing a 15 mJ pulse into an argon gas cell. The HHG source was imaged onto the amplifier using a grazing-incidence toroidal mirror, and the 25th harmonic could be tuned to the lasing transition by chirping the driver beam. Due to collisional ionization of the lasing ions, the gain had a short lifetime and was strongly peaked^31^. The time delay between the creation of the amplifier and seeding was set at the experimentally obtained value of 1.2 ps to match the peak of strongest amplification. After exiting the laser-plasma amplifier and passing through two aluminium filters blocking residual NIR light, the XUV pulses were focused onto the sample by two spherical multilayer mirrors with 5 m (mirror 2) and 0.5 m (mirror 1) focal lengths. This telescope resulted in a demagnification of 10. To reduce aberration and match the multilayer conditions, the angle of incidence on the curved mirrors was minimized to 4°. To implement ptychographic scanning, the sample was mounted on a 3D positioner with a repeatability of 50 nm over the complete travel range of 30 mm. The intensity distribution of diffraction patterns was recorded with a 2048 × 2048 pixel CCD detector with a 16 bit dynamic range and a 27.8 mm diagonal size (Andor iKon-L SO). The camera was cooled to −50 °C to reduce electronic noise. Since the sample was 55 mm from the camera, the numerical aperture of our system (0.25) set the resolution limit to ~65 nm.

### Sample

The sample, mounted in the focal plane of mirror 1, consisted of a gold-coated carbon grid with an overall thickness of 50 nm with a regular hole pattern of 1 µm in hole diameter and 2 µm in pitch (see Fig. 1b).

### Ptychographic reconstruction

To retrieve the complex-valued wavefront at the plane of the sample, a ptychographic approach was employed. The sample was scanned with respect to the XUV beam in a spiral pattern to avoid so-called grid-pathology artefacts^16^, with 30 scan points in total. For a successful reconstruction, the overlap between neighbouring scan points has to be fairly high. In consideration of the total amount of exposure, an overlap of 90% was chosen. To obtain a sufficient scan map, a rough estimate of the coherent focal spot size is necessary. A reconstruction of a single diffraction pattern of the sample obtained using the Hybrid-Input-Output algorithm^32^ with a feedback parameter of *β* = 0.9 was used to evaluate the rough size of the spot by counting the reconstructed holes resulting in a 5.6 ± 0.2 µm FWHM (see Supplementary S3). Using the ePIE algorithm^18^ with 3000 iterations and a 6-µm diameter flat-top initial probe, the coherent complex-valued object and illumination functions were retrieved with a resolution of approximately 200 nm. The feedback parameters were chosen to be *α* = *β* = 0.9. A delay between object and probe reconstruction of 10 iterations was introduced to avoid artefacts from oscillations between the object and probe field. To compensate for beam drifts and variations, the measurement and reconstruction were repeated five times and averaged.

## Backpropagation

The used optics and beam path length allow backpropagation to the exit plane of the plasma channel using the angular spectrum propagation method (see supplementary materials section S1). Here, mirrors were applied as phase shifters, and the incident angles were considered to compensate for possible coma aberration. Due to the difference between the fields-of-view (FOVs) of the probe field (220 µm) and exit wave (2200 µm), the FOV was adapted via zero padding and oversampling during propagation to ensure a sufficient bandwidth. To allocate the exit wave in the last part of the propagation from mirror 2 to the source, the beam was propagated in slices of 10 mm to check the position of the focal spot. Assuming the exit wave location matches the focal spot of mirror 2, the backpropagation ends if the exit wave reaches its most co form.

## Simulations

The creation of the plasma channel was modelled with the 2D radiative hydrodynamics code *ARWEN*^26^. Particle-In-Cell codes (*WAKE-EP*^24^ and *Calder-Circ*^25^) were used to model the propagation of the NIR pulse throughout the plasma channel^20^. The resulting 3D electron density and Kr^8+^ abundance were fed to our 3D Maxwell–Bloch code *Dagon*^21^. The atomic data and collisional rates fed to *Dagon* were obtained from the code *OFIKinRad*^22^. With *Dagon*, we modelled the amplification of a high-order harmonic throughout a 2 mm inhomogeneous plasma amplifier. This is the effective amplification length given by our PIC simulations. Since our Maxwell–Bloch model gives the complex-valued electric field of the amplified pulse, its intensity and phase profiles can be directly compared to propagated ptychographic measurements.

## Supplementary information

Supplementary Materials

## References

[1] Helander P (2012). Stellarator and tokamak plasmas: a comparison. Plasma Phys. Control. Fusion.

[2] Li Z (2019). Experimental investigation of Z-pinch radiation source for indirect drive inertial confinement fusion. Matter Radiat. Extrem.

[3] Rohringer N (2012). Atomic inner-shell X-ray laser at 1.46 nanometres pumped by an X-ray free-electron laser. Nature.

[4] Yoneda H (2015). Atomic inner-shell laser at 1.5-ångström wavelength pumped by an X-ray free-electron laser. Nature.

[5] Depresseux A (2015). Table-top femtosecond soft X-ray laser by collisional ionization gating. Nat. Photonics.

[6] Zürch M (2017). Transverse coherence limited coherent diffraction imaging using a molybdenum soft X-ray laser pumped at moderate pump energies. Sci. Rep..

[7] Spielmann CH (1997). Generation of coherent X-rays in the water window using 5-femtosecond laser pulses. Science.

[8] Cayzac W (2017). Experimental discrimination of ion stopping models near the Bragg peak in highly ionized matter. Nat. Commun..

[9] Vinko SM (2015). Investigation of femtosecond collisional ionization rates in a solid-density aluminium plasma. Nat. Commun..

[10] He Z-H (2015). Coherent control of plasma dynamics. Nat. Commun..

[11] Sullivan JV, Walsh A (1965). High intensity hollow-cathode lamps. Spectrochim. Acta.

[12] Wagner C, Harned N (2010). Lithography gets extreme. Nat. Photonics.

[13] Legall H (2012). Compact X-ray microscope for the water window based on a high brightness laser plasma source. Opt. Express.

[14] Silfvast WT (1999). Intense EUV incoherent plasma sources for EUV lithography and other applications. IEEE J. Quantum Electron.

[15] Schmitz C (2016). Compact extreme ultraviolet source for laboratory-based photoemission spectromicroscopy. Appl. Phys. Lett..

[16] Thibault P, Dierolf M, Bunk O, Menzel A, Pfeiffer F (2009). Probe retrieval in ptychographic coherent diffractive imaging. Ultramicroscopy.

[17] Sebban S (2002). Demonstration of a Ni-like Kr optical-field-ionization collisional soft X-Ray laser at 32.8 nm. Phys. Rev. Lett..

[18] Maiden AM, Rodenburg JM (2009). An improved ptychographical phase retrieval algorithm for diffractive imaging. Ultramicroscopy.

[19] Vartanyants IA (2011). Coherence properties of individual femtosecond pulses of an X-ray free-electron laser. Phys. Rev. Lett..

[20] Oliva E (2018). Hydrodynamic evolution of plasma waveguides for soft-x-ray amplifiers. Phys. Rev. E.

[21] Oliva, E. et al. DAGON: a 3D Maxwell-Bloch code. In *SPIE Proceedings Volume 10243, X-ray Lasers and Coherent X-ray Sources: Development and Applications* (eds. Klisnick, A. & Menoni, C. S.) (SPIE, 2017).

[22] Cros B (2006). Characterization of the collisionally pumped optical-field-ionized soft-x-ray laser at 41.8 nm driven in capillary tubes. Phys. Rev. A.

[23] Oliva E (2015). Self-regulated propagation of intense infrared pulses in elongated soft-x-ray plasma amplifiers. Phys. Rev. A.

[24] Paradkar BS, Cros B, Mora P, Maynard G (2013). Numerical modeling of multi-GeV laser wakefield electron acceleration inside a dielectric capillary tube. Phys. Plasmas.

[25] Lifschitz AF (2009). Particle-in-cell modelling of laser–plasma interaction using Fourier decomposition. J. Comput. Phys..

[26] Ogando F, Velarde P (2001). Development of a radiation transport fluid dynamic code under AMR scheme. J. Quant. Spectrosc. Radiat. Transf..

[27] Carlström S, Mauritsson J, Schafer KJ, L’Huillier A, Gisselbrecht M (2017). Quantum coherence in photo-ionisation with tailored XUV pulses. J. Phys. B. Mol. Opt. Phys..

[28] Wituschek A (2020). Tracking attosecond electronic coherences using phase-manipulated extreme ultraviolet pulses. Nat. Commun..

[29] Loriot V (2015). Resolving XUV induced femtosecond and attosecond dynamics in polyatomic molecules with a compact attosecond beamline. J. Phys. Conf. Ser..

[30] Durfee CG, Lynch J, Milchberg HM (1995). Development of a plasma waveguide for high-intensity laser pulses. Phys. Rev. E.

[31] Zeitoun P (2004). A high-intensity highly coherent soft X-ray femtosecond laser seeded by a high harmonic beam. Nature.

[32] Fienup J (1987). Reconstruction of a complex-valued object from the modulus of its Fourier transform using a support contraint. Opt. Soc. Am..

[33] Jaeglé, P. *Coherent Sources of XUV Radiation* (Springer International Publishing, 2006).

