# Supplementary material for: Nonlinear ionization dynamics of hot dense plasma observed in a laser-plasma amplifier
Source: Light Sci Appl. 2020 Nov 18;9:187. doi: 10.1038/s41377-020-00424-2 (PMC7673011; doi:10.1038/s41377-020-00424-2)
Supplement: Supplementary file 1 — Supplementary Materials [file 41377_2020_424_MOESM1_ESM.pdf]

Supplementary Information

Nonlinear Ionization Dynamics of Hot Dense Plasma Observed in a Laser Plasma Amplifier

F. Tuitje[1,2], P. Martínez Gil[3], T. Helk[1,2], J. Gautier[4], F. Tissandier[4], J.-P.Goddet[4], A. Guggenmos[5,6], U. Kleineberg[5], S. Sebban[4], E. Oliva[3], C. Spielmann[1,2], and M. Zürch[1,2,7,8,9]

[1] Institute for Optics and Quantum Electronics, Abbe Center of Photonics, University of Jena, Jena, Germany
[2] Helmholtz Institute Jena, Jena, Germany
[3] Departamento de Ingeníeria Energética, ETSI Industriales, Universidad Politécnica de Madrid, Instituto de Fusión Nuclear “Guillermo Velarde”, Universidad Politecnica de Madrid, Madrid, Spain
[4] Laboratoire d'optique appliquée, ENSTA-ParisTech, Palaiseau, France
[5] Department for Physics, Ludwig-Maximilian-University Munich, Garching, Germany
[6] UltraFast Innovations GmbH, Garching, Germany
[7] Fritz Haber Institute of the Max Planck Society, Berlin, Germany
[8] University of California at Berkeley, Department of Chemistry, Berkeley, USA
[9] Lawrence Berkeley National Laboratory, Materials Sciences Division, Berkeley, USA

S1 Angular spectrum propagation of the reconstructed probe

To propagate the reconstructed complex-valued illumination function back to its source, a bandwidth limited angular spectrum propagator was used[1]. The mirrors were applied as a curved phase shift overlaid with a tilted plane under a reflection angle α

$$\Phi_M = \exp\left(i\frac{2\pi}{\lambda}\sqrt{r^2-u^2-v^2}\right)\exp\left(i\frac{2\pi}{\lambda}\tan(\alpha)\,u\right) \tag{1}$$

onto the propagated field Φ_{in} via an elementwise complex Hadamard product

$$\Phi_{out} = \mathfrak{P}(\Phi_{in}, d) \odot \Phi_M \tag{2}$$

$\mathfrak{P}$ assign the angular spectrum propagator to

$$\mathfrak{P}(\Phi_{out}, d) = \mathfrak{F}^{-1}\left(\mathfrak{F}(\Phi_{in}) \odot \exp\left(i\frac{2\pi}{\lambda}d + i\pi\lambda d(u^2+v^2)\right)\right) \tag{3}$$

with $\mathfrak{F}$ and $\mathfrak{F}^{-1}$ as the Fourier resp. inverse Fourier transform, λ as the wavelength, d as the propagation distance and u, v as the pixel coordinates of the fields with a centered origin. Further propagation via mirror 2 to the position of the exit wave completes the path of propagation to:

$$\Phi_{exit} = \mathfrak{P}(\mathfrak{P}(\mathfrak{P}(\Phi_{probe}, d_1) \odot \Phi_{M1}, d_2) \odot \Phi_{M2}, d_3) \tag{4}$$

Due to the increasing beam waist with propagation to the first mirror, the probe field Φ_{in} was zero-padded to increase the field-of-view to 1 cm to avoid information loss and aliasing.

S2 Evaluation of the exit wave's position

The actual position of the exit wave may not exactly agree with the focal distance d_3 of mirror 2 due to experimental variances. Hence, the numerical distances were slightly varied to find the focus within an offset distance $\delta = 1\,m$, varying from $d_3 - \frac{\delta}{2}$ to $d_3 + \frac{\delta}{2}$. By extracting equidistant slices of Φ_{exit} in $d_3 \pm \frac{\delta}{2}$ for every 10 mm, a focal cross section was determined.

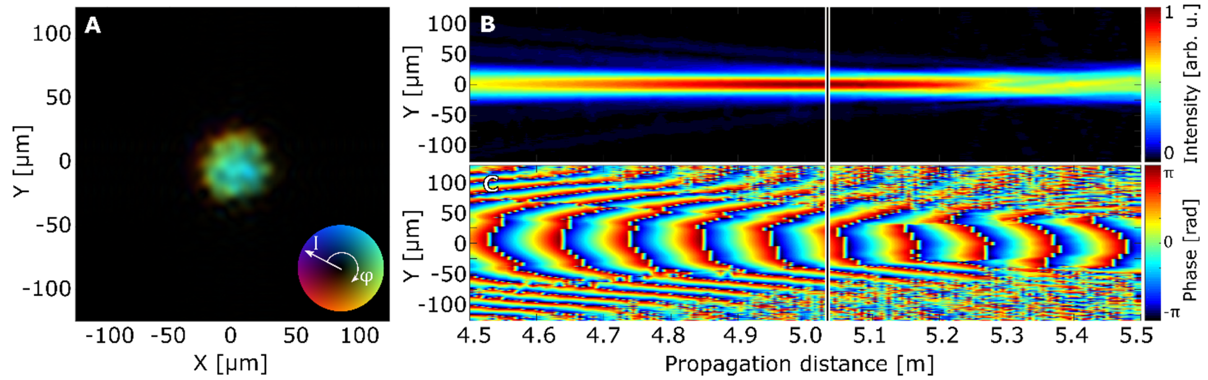

Fig. S1: Focal cross section around d_3. The last propagation d_3 to the exit wave Φ_{exit} (**A**) was varied with $\delta = 1\,m$ to find the physical focus via an intensity (**B**) and phase (**C**) cross section. Here the flip of sign of phase curvature (white/black line in **B**,**C**) was used as a criterion together with the position of highest intensity in (**B**). In (**C**), the visible period of ~10 cm represents not the wavelength, rather it is formed by aliasing due to the slice-to-slice distance.

Figure S1**B**,**C** show the focal point in phase and intensity of the numerical propagation after mirror 2, which is ~35 mm shifted along the beam direction with respect to the mirror defined focal length of 5 m. The final exit wave is shown in Figure S1**A** and is used for the comparison to the simulation.

S3 Focus size and beam stability

Creating the ptychographic scan map requires a rough estimate of the coherent spot size in the sample plane[2,3]. We used a CDI reconstruction of a single-shot diffraction pattern of the sample (Quantifoil Micro Tools GmbH, Quantifoil® R1/2) to investigate the spot size as the reconstruction can be considered as the product between the illuminating field and the sample itself. After the reconstruction, a 2D Gaussian function can be fitted to the reconstructed structure, as seen in Fig. S2. In this way, the coherent part of the total illumination in the sample plane can be read out to 5.6 ± 0.2 µm FWHM.

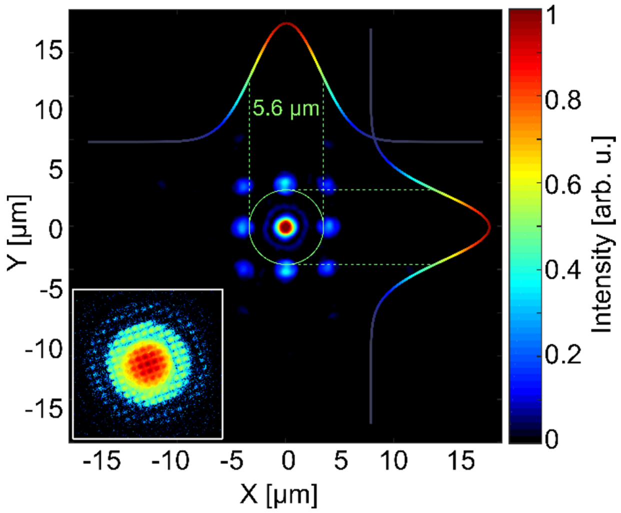

Fig. S2: Reconstructed periodic hole sample with corresponding diffraction pattern (inset). By fitting a 2D gaussian function to the reconstruction, the coherent width (FWHM) of the illumination function is estimated to be ~5.6 µm. The aspect ratio is ~1.

Ptychography without sophisticated techniques as e.g. mixed state reconstruction[4] or map refinement[5], requires spatially stable beams and low shot-to-shot variations of the wave fronts. We analyzed variations between consecutive shots by recording 100 single-shot diffraction patterns of an isolated object with an overall size of ~5 µm. Correlating the shots to a far-field simulation of the object revealing possible beam jitter. Here, a limit of correlation of 0.75 was set to separate stable diffraction patterns from unstable ones. ~40 % of the recorded pattern can be considered as stable and, therefore, ~40 % of shots can be considered as jitter free. For this reason, the measurement of the main dataset was repeated several times to ensure stable results for every scanning position.

S4 Discussion of other plasma detection methods

Interferometry is capable to determine the electron density in a plasma by measuring the phase shift of a well-timed coherent probe beam. After an Abel inversion of the recorded interference pattern the refraction index and electron density can be reconstructed[6–8]. The smallest resolvable features are limited by the size and contrast of interference fringes. Shadowgraphy, where light traverses the plasma and is recorded afterwards, requires a less sophisticated setup but is more limited regarding its quantitative output. Out of the resulting contrast, the refraction index and absorption can be inferred[9]. This method also requires an Abel inversion and is, therefore, limited to radial-symmetric homogenous plasmas. A full characterization of the ionization states in their spatial distribution is hardly realizable[10]. Other methods, like spectroscopy[11] and Thomson scattering[12] can unveil the ionic populations inside the plasma. However, they also require complex experimental setups and the support from modelling to correctly understand the results.

S5 Modelling of the plasma evolution and amplification process

The generation of a plasma amplifier and its posterior interaction with a high harmonic seed is a multiscale problem that requires advanced codes that cover all the characteristic scales involved, from the nanosecond hydrodynamic expansion of the plasma waveguide to the picosecond collisional processes inside the plasma and finally to the femtosecond duration of Rabi oscillations in the amplified pulse. We have used a multiphysics set of codes allowing us to study all the phenomena involved in this SXRL.

The creation and evolution of the plasma waveguide has been modelled with the 2D radiation-hydrodynamics code ARWEN[13]. By taking advantage of the cylindrical symmetry of the problem we modelled the focal line of the axicon lens using the ray-tracing capabilities of the code. The resulting expansion velocity and electron density profile allowed us to assess the experimental results[14].

The electron density radial profile of the plasma waveguide was fed to particle-in-cell codes, WAKE-EP[15] and Calder-Circ[16]. With these codes we modelled the propagation of the intense IR pump pulse through the plasma waveguide and the creation of the lasing ion. Oscillatory (focusing-defocusing) propagation dynamics[17] and overionized regions[14] along the plasma were observed.

The threshold intensity to obtain the ion Kr^{8+} by optical field ionization is approximately $I \sim 10^{17}$ W cm^{-2}. This is the intensity that is best propagated throughout the waveguide (PIC simulations show the waveguide presents strong loses in the first millimeter), but several effects increase it in some parts of the amplifier. This intensity is taken as an input to our 0D collisional-radiative code, OFIKinRad[18], used to compute the temporal dynamics of the atomic processes that take place in the plasma, i.e. collisional excitation and deexcitation, radiative decay, non-equilibrium ionic populations, gain, and others (see Ref. 18 for details).

Finally, we use our 4D Maxwell-Bloch code, Dagon[19], to model the amplification of the seed beam throughout the plasma. The electron density and lasing ion profiles are imported from our PIC simulations. From the more than 90 ionic levels used in the atomic computation we select a few of them to be fed, alongside the corresponding collisional rates, to Dagon. This way, the population inversion computed in Dagon is sufficiently accurate. The intensity and phase of the amplified beam are directly computed from the real and imaginary parts of the electric field, directly available from Dagon.

S6 Shaping the intensity and phase profiles of the amplified beam

As it has been shown in the main text, the plasma amplifier inhomogeneities, both in electron and lasing ion densities, imprint the intensity and wavefront profiles of the amplified seed. This effect paves the way towards tailoring the resulting amplified beam profile on demand, both in intensity and phase. While the effect highlighted in the main text, the impact of the spatial abundance of the lasing ion in the intensity and phase profile, is difficult to control experimentally, there are other parameters that can be adjusted to modify the profiles. Among them, the amplifier length is relatively easy to vary and has a strong impact in the amplified beam.

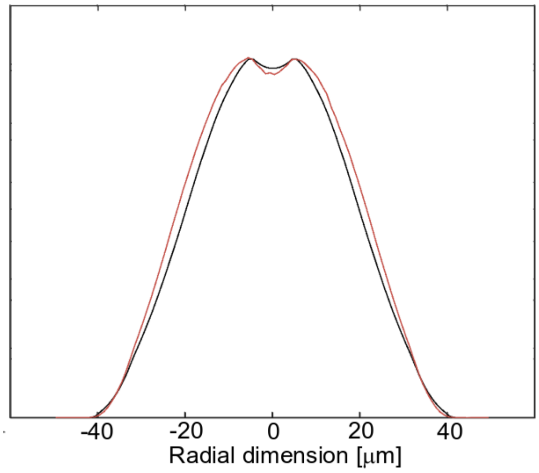

Fig. S3: Radial intensity profile for an amplification length of 2 mm (red) and 3 mm (black).

Figure S3 shows the radial intensity profile for two different amplification lengths, 2 mm (red) and 3 mm (black). While the overall shape is similar in both cases (a localized central dip caused by the overionization in the amplifier and a Gaussian profile given by the lasing ion abundance) there are some differences. First, the central dip is less pronounced in the 3 mm case, the stronger amplification tends to homogenize the profile. Second, while the radial Gaussian decay is similar in both cases, there are differences in the wings that can be tentatively correlated with the experimental results shown in Figure 2 in the main text. In that figure, the radial intensity profile can be divided by two regions adjusted by two gaussians. The first one is the central part, with a diameter of approximately 30 micrometers and the second one comprises the rest of the radial profile. This can be explained as follows: the central part of the amplifier is optimally pumped and the amplification length there is larger than in the lateral region, where the intensity of the pumping IR pulse can decay after some millimeters of propagation, reducing the effective amplification length, thus explaining the shape of the intensity profile.

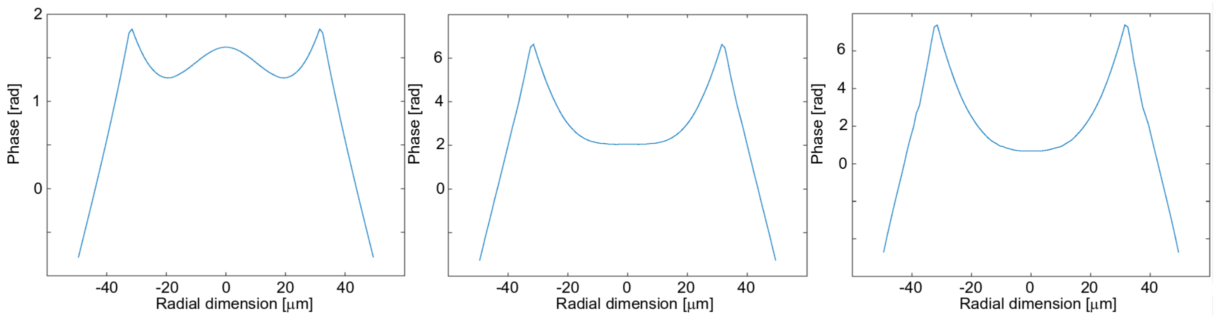

Fig. S4: Radial phase profile for three different lengths. (left) 1 mm, (center) 4 mm, (right) 5 mm. The amplifier has two overionized regions, one in the first millimeter and the second in its center.

The amplification length has a stronger effect in the phase radial profile. Figure S4 shows the phase at three different propagation lengths, 1 mm (left), 4 mm (center) and 5 mm (right). The

amplifier, which in this case is a 5 mm plasma waveguide, presents two overionized regions, one in the first millimeter of the plasma and the second one in the middle. As it is shown in Figure S4 left, the increased electron density due to the overionization strongly impacts the shape of the phase. The channel shape is not retrieved and instead an inverted parabola appears. After some amplification, the phase profile changes and at 4 mm it presents a flat-top profile in the central part, shown in Figure S4 center. Further amplification in the waveguide modifies the phase profile towards the parabolic shape induced by the plasma waveguide.

These results show the possibility of controlling the shape of the beam, both in intensity and wavefront. Since there are several parameters that can be varied experimentally (e.g., amplifier length, plasma waveguide width, pump laser intensity), our experimental and modelling results pave the way towards delivering amplified beams with on-demand wavefronts. These tailored beams could have a strong impact in fields as coherent diffraction imaging and ultrafast holography.

References

1. Delen, N. & Hooker, B. Free-space beam propagation between arbitrarily oriented planes based on full diffraction theory: a fast Fourier transform approach. *JOSA A* **15**, 857–867 (1998).
2. Maiden, A. M. & Rodenburg, J. M. An improved ptychographical phase retrieval algorithm for diffractive imaging. *Ultramicroscopy* **109**, 1256–1262 (2009).
3. Bunk, O.; Dierolf, M.; Kynde, S.; Johnson, L.; Marti, O.; Pfeiffer, F. Influence of the overlap parameter on the convergence of the ptychographical iterative engine. *Ultramicroscopy* **108**, 481–487 (2007).
4. Thibault, P. & Menzel, A. Reconstructing state mixtures from diffraction measurements. *Nature* **494**, 68–71 (2013).
5. Dwivedi, P., Konijnenberg, A. P., Pereira, S. F. & Urbach, H. P. Lateral position correction in ptychography using the gradient of intensity patterns. *Ultramicroscopy* **192**, 29–36 (2018).
6. Bukin, V. V., Garnov, S. V., Malyutin, A. A. & Strelkov, V. V. Interferometric diagnostics of femtosecond laser microplasma in gases. *Phys. Wave Phenom.* **20**, 91–106 (2012).
7. Cao, L. *et al.* Space-time characterization of laser plasma interactions in the warm dense matter regime. *Laser Part Beams 2520072239-244* **25**, (2008).
8. Sävert, A. *et al.* Direct Observation of the Injection Dynamics of a Laser Wakefield Accelerator Using Few-Femtosecond Shadowgraphy. *Phys. Rev. Lett.* **115**, 055002 (2015).
9. Gopal, A., Minardi, S. & Tatarakis, M. Quantitative two-dimensional shadowgraphic method for high-sensitivity density measurement of under-critical laser plasmas. *Opt. Lett.* **32**, 1238–1240 (2007).
10. Schnell, M. *et al.* Deducing the Electron-Beam Diameter in a Laser-Plasma Accelerator Using X-Ray Betatron Radiation. *Phys. Rev. Lett.* **108**, 075001 (2012).
11. Glenzer, H. S. *et al,* Ionization Balance in Inertial Confinement Fusion Hohlraums, *Phys. Rev. Lett.* **87**, 4, 045002, (2001)
12. Glenzer, H. S. X-ray Thomson scattering in high energy density plasmas, *Rev. Mod. Phys.* **81**, (2009)
13. Ogando, F. and Velarde, P. Development of a radiation transport fluid dynamic code under AMR scheme. *J. Quant. Spectrosc. Radiat. Transf.* **71**, 541-550 (2001)
14. Oliva, E. *et al,* Hydrodynamic evolution of plasma waveguides for soft-x-ray amplifiers, *Phys. Rev. E* **97**, 023203 (2018)
15. Paradkar, B. *et al,* Numerical modelling of multi-GeV laser wakefield electron acceleration inside a dielectric capillary tube, *Phys. Plasmas* **20**, 083120 (2013)
16. Lifshitz, A. F. *et al,* Particle-In-Cell modelling of laser-plasma interaction using Fourier decomposition, *J. Comput. Phys.* **228**, 1803-1814 (2009)
17. Oliva, E. *et al*, Self-regulated propagation of intense infrared pulses in elongated soft-x-ray plasma amplifiers, *Phys. Rev. A* **92**, 023848 (2015)

18. Cros, B. *et al*, Characterization of the collisionally pumpued optical-field-ionized soft-x-ray laser at 41.8 nm driven in capillary tubes, *Phys. Rev. A* **73**, 033881 (2006)
19. Oliva, E. *et al*, DAGON: a 3D Maxwell-Bloch code, in *SPIE Proceedings Volume 10243, X-ray lasers and Coherent X-ray Sources: Development and Applications (eds.Klisnick, A. & Menoni, C. S.)* 1024303 (2017)
